# EpiTracer - an algorithm for identifying epicenters in condition-specific biological networks

**DOI:** 10.1186/s12864-016-2792-1

**Published:** 2016-08-18

**Authors:** Narmada Sambaturu, Madhulika Mishra, Nagasuma Chandra

**Affiliations:** 1 IISc Mathematics Initiative, Indian Institute of Science, Bangalore, 560012 India; 2 Department of Biochemistry, Indian Institute of Science, Bangalore, 560012 India

**Keywords:** Network mining, Influential nodes, Ripple centrality, Perturbation analysis, Condition-specific network

## Abstract

**Background:**

In biological systems, diseases are caused by small perturbations in a complex network of interactions between proteins. Perturbations typically affect only a small number of proteins, which go on to disturb a larger part of the network. To counteract this, a stress-response is launched, resulting in a complex pattern of variations in the cell. Identifying the key players involved in either spreading the perturbation or responding to it can give us important insights.

**Results:**

We develop an algorithm, EpiTracer, which identifies the key proteins, or epicenters, from which a large number of changes in the protein-protein interaction (PPI) network ripple out. We propose a new centrality measure, ripple centrality, which measures how effectively a change at a particular node can ripple across the network by identifying highest activity paths specific to the condition of interest, obtained by mapping gene expression profiles to the PPI network.

We demonstrate the algorithm using an overexpression study and a knockdown study. In the overexpression study, the gene that was overexpressed (PARK2) was highlighted as the most important epicenter specific to the perturbation. The other top-ranked epicenters were involved in either supporting the activity of PARK2, or counteracting it. Also, 5 of the identified epicenters showed no significant differential expression, showing that our method can find information which simple differential expression analysis cannot. In the second dataset (SP1 knockdown), alternative regulators of SP1 targets were highlighted as epicenters. Also, the gene that was knocked down (SP1) was picked up as an epicenter specific to the control condition. Sensitivity analysis showed that the genes identified as epicenters remain largely unaffected by small changes.

**Conclusions:**

We develop an algorithm, EpiTracer, to find epicenters in condition-specific biological networks, given the PPI network and gene expression levels. EpiTracer includes programs which can extract the immediate influence zone of epicenters and provide a summary of dysregulated genes, facilitating quick biological analysis. We demonstrate its efficacy on two datasets with differing characteristics, highlighting its general applicability. We also show that EpiTracer is not sensitive to minor changes in the network. The source code for EpiTracer is provided at Github (https://github.com/narmada26/EpiTracer).

## Background

A biological system consists of a large number of proteins involved in a series of intricate and tightly orchestrated interactions. Representing this complex system as a network allows us to harness network-mining methodologies to analyse the system as a whole. Diseases typically affect only a small number of proteins [1, 2]. The immediate interacting partners of these proteins can be expected to show a change in expression levels or behavior. In addition, the inter-connected nature of the system causes cascade effects, altering the levels of proteins far removed from the original source. At the same time, the system may attempt to restore its equilibrium by launching a stress-response [3]. It would be interesting and useful to identify the key players in this tug-of-war, which are most influential in either spreading or curtailing the perturbation. These key proteins are referred to as epicenters specific to that condition.

A vast amount of data is generated by microarray experiments, which provide a snapshot of the active and inactive players of the system. These datasets are available on public databases such as Omnibus [4]. Most studies in biology focus on only a few proteins or pathways, and work with a restricted field of view. Through algorithms such as EpiTracer, we hope to enable the analysis of large scale and detailed models, giving a picture which reflects the intricate workings of living systems more closely. In this paper, we work with a dataset consisting of nearly half the complement of human genes.

In this paper, we develop an algorithm called EpiTracer, which identifies the epicenters from which either the perturbation or the reaction to it ripples out. This is done using a protein-protein interaction (PPI) network into which gene expression levels before and after the perturbation are integrated. To the best of our knowledge, no method exists currently which can identify epicenters with this type of data. Other methods that provide insights into influential nodes require a causal network as input, where each edge depicts a causal relationship, and is directed from the cause to the effect [5, 6]. However, clear-cut causal dependencies have been established for only a small set of proteins, making it impossible to analyse large networks. Network motifs have also been used to highlight important proteins in directed biological networks [7]. However these methods do not make use of information about changes in expression levels of genes, thus losing out on a rich source of information. Methods also exist which highlight the nodes which, when intentionally perturbed, spread the perturbation the fastest [8]. This is not the same as identifying the epicenter of a naturally occurring perturbation, which is a more complicated and biologically relevant scenario.

The EpiTracer algorithm is based on the observation that an epicentric protein would have to be highly active in order to exert its influence, and also have good connectivity in order for its influence to spread. We define a new centrality measure called *ripple centrality*, which gives a combined measure of a node ^′^s activity as well as its connectivity, thus allowing us to rank proteins on their ability to be an effective epicenter. The top-ranked proteins qualify to be epicenters. The algorithm combines the PPI network and gene expression levels in such a way as to ease the computation of active paths. The sub-network with high activity paths only in the perturbed condition is extracted, thus reducing the search space for the next step. The nodes in this sub-network are then ranked on the basis of their ripple centrality score, with the top 10 nodes considered as epicenters. The efficacy of the algorithm is demonstrated through two case studies. The first case study analyses human glioma cell line (U251) upon overexpression of the gene PARK2 (GSE61973) [9]. The algorithm was able to identify PARK2 as the most important epicenter without any prior knowledge of the perturbation. Functional enrichment analysis showed that most of the top 10 epicenters play a role in enabling or countering the activity of PARK2. Also, 5 of the top 10 epicenters showed no significant fold change, proving that our method is capable of identifying more than simple differential expression analysis. The EpiTracer pipeline includes a program for extracting the immediate influence zone of the epicenters. Analysis of the immediate influence zone of the top-ranked epicenter (PARK2) showed that it was enriched in genes involved in cell-cycle regulation. The second case study attempts to identify the target genes regulated by transcription factor SP1 by knocking down the expression of SP1 in HeLa cells (GSE37935) [10]. In this study, EpiTracer was able to identify SP1 among the top ranked epicenters. Sensitivity analysis was carried out by increasing the gene expression levels of all nodes by upto 5 % (100 independent experiments), and decreasing the gene expression levels of all nodes by upto 5 % (100 independent experiments). It was found that irrespective of the direction or extent of perturbation, 9 nodes always appear in the top 10 ranks, and 16 nodes always appear in the top 20 ranks of epicenters. This shows that the nodes ranked as epicenters remain largely unaffected even when every gene in the system is subjected to a minor change.

## Methods

A high-density protein-protein interaction network was reconstructed for use in this work. Condition-specific gene expression profiles were obtained from published literature. The inputs as well as the algorithm are explained below.

### Protein-protein interaction network

A base network containing known and predicted protein-protein interactions, genetic interactions and regulatory interactions with directions was taken from Khurana et. al., 2013 [11]. Metabolic interactions from KEGG [12] were added to this, resulting in a directed network with 10,306 nodes and 74,404 edges.

### Gene expression profiles

Two gene expression datasets were obtained from the GEO database [13]. In the first dataset GSE61973 [9], PARK2 gene was overexpressed in human glioma cell line (U251). In the second study GSE37935 [10], SP1 gene was knocked down using siRNA in HeLa cells. These two case studies were selected to demonstrate the general applicability of the EpiTracer algorithm. GeneSpringX 12.6.1, with Robust Multichip Averaging (RMA) [14] was used for microarray data normalization. A 1.5 fold cut-off was applied for differential gene expression analysis (P-value ≤ 0.05 by T-test with Benjamini-Hochberg false discovery rate correction).

### Combining the inputs

**Fig. 1:**
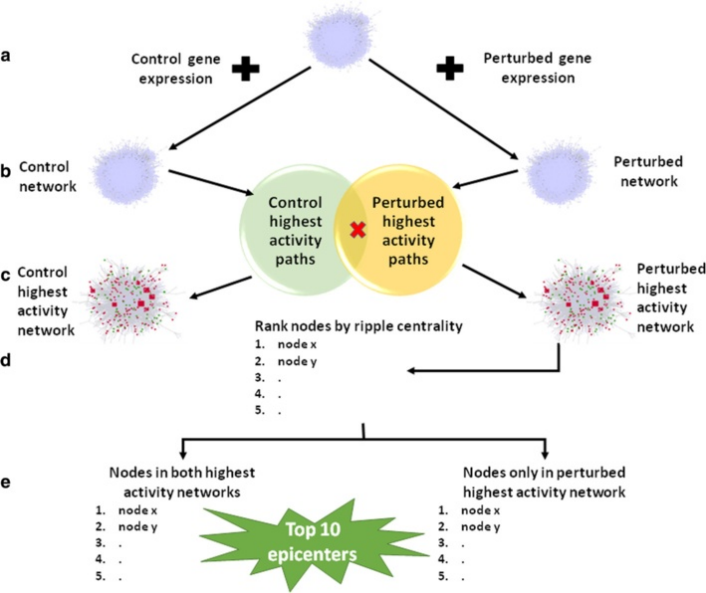
The EpiTracer workflow. **a** Gene expression profiles of each condition are mapped onto the base PPI network. **b** Highest activity paths are calculated for each condition, and common paths are discarded, giving condition-specific highest activity paths (CSHAPs). **c** The network induced by the CSHAPs form the condition-specific highest activity networks (CSHANs). **d** Nodes in the perturbed highest activity network are ranked according to ripple centrality. **e** The ranked list of nodes is split into two lists based on overlaps with the control highest activity network. Top 10 nodes in the list unique to the perturbed condition form the epicenters specific to the perturbation

The gene expression profile of each condition was mapped onto the PPI network, to create one weighted network per condition (Fig. 1(a)). The nodes (proteins) were given a weight equal to the normalized signal intensity for the corresponding gene in that condition. ${w_{i}^{x}} = SI^{x}$ where ${w_{i}^{x}}$ is the weight of node *i* in condition *x*, and *SI*^*x*^ is the normalized signal intensity in condition *x*. This formulation stems from the assumption that the expression level of a gene gives a reasonably good approximation of the abundance of the protein in the system. The cost of an edge (protein-protein interaction) was taken as a function of the abundance of the participating proteins, as

$$ {c_{i}^{x}} = \frac{1}{\sqrt{{w_{u}^{x}} * {w_{v}^{x}}}} $$

where ${c_{i}^{x}}$ is the cost of edge *i* in condition *x*, and ${w_{u}^{x}}$, ${w_{v}^{x}}$ are the weights of the nodes comprising the edge. This follows from the assumption used in mass-action kinetics, that the activity of a reaction is directly proportional to the concentration of the participants. Taking the inverse makes sure that a highly active interaction has a very low edge cost.

Given a path with *n* edges, the sum of costs of the edges involved in the path gives the cost of the path.

$$ pathcost = \sum_{i=1}^{n}{c_{i}^{x}} $$

where ${c_{i}^{x}}$ is the edge cost for each edge in the path, and *n* is the length of the path. A shortest path algorithm will preferentially choose edges with the least cost for a given source and destination, which in our formulation translates to identifying the highest activity path.

### EpiTracer algorithm – rationale

In order to be effective, an epicenter should be highly active and participate in high activity paths only in the perturbed condition. To capture this, we calculate highest activity paths in each condition and discard common paths. The common paths correspond to the paths which remain highly active and unchanged irrespective of the perturbation. Such paths add no information about the perturbation (Fig. 1(b)). The edges involved in these CSHAPs induce a sub-network of the original network, referred to as the condition-specific highest activity networks (CSHANs) (Fig. 1(c)).

An epicenter should also be able to reach many nodes in the network in order to exert its influence, and the paths from the epicenter to these nodes must also be highly active. This is captured by the new centrality measure proposed here termed *ripple centrality*, and is explained below.

#### Closeness centrality

**Fig. 2:**
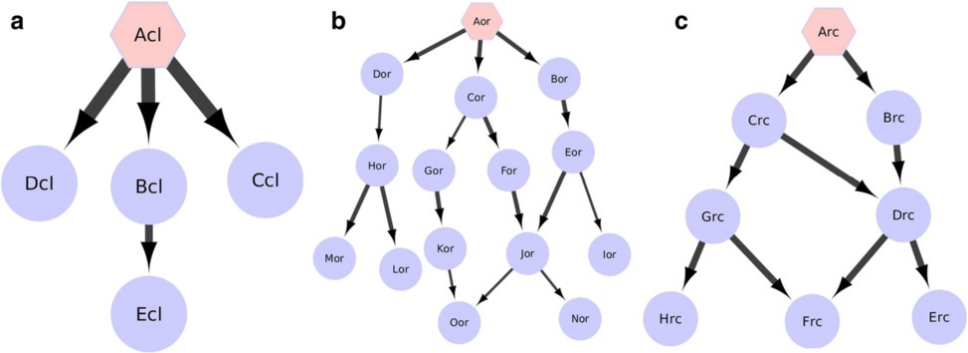
Illustration of ripple centrality. **a** Node Acl is the source of highly active paths, and has high closeness centrality. However it can only reach 4 nodes, and is not a good epicenter. **b** Node Aor can reach 14 nodes, but paths originating at Aor have low activity. Thus it is not a good epicenter. **c** Node Arc is the source of highly active paths and can reach a large number of nodes (7), making it the best candidate for an epicenter. The hexagon represents candidate epicenters

Closeness centrality [15] of a node *u* is defined as the reciprocal of the sum of shortest path costs from *u* to every reachable node *v*

$$ C(u) = \frac{1}{\sum_{v}\sigma(u,v)} $$

where *σ*(*u,v*) is the cost of the shortest path from *u* to *v*. Because of the way edge costs are formulated, a node *u* with highly active paths to a set of nodes *v* will have high closeness centrality. This is depicted by node Acl in Fig. 2a. Here a thicker edge corresponds to a highly active reaction.

#### Outward reachability

Given a node *u*, the number of nodes reachable from *u* is termed its outward reachability [16].

$$ R_{out}(u) = \left\vert{nodes\, reachable\, from\, u}\right\vert $$

where *R*_*out*_(*u*) denotes outward reachability of *u*.

#### Ripple centrality

In Fig. 2a, the node Acl represents nodes which have very high activity paths, but to only a small number of nodes. Such a node would have high closeness centrality [15], but would not be a good candidate for an epicenter as any perturbation arising at this point could not spread to a large number of nodes. On the other hand, node Aor (Fig. 2b) represents nodes which have very good connectivity, but participate in relatively low activity paths. These types of nodes would have high outward reachability, but are poor candidates for epicenters. Thus neither closeness centrality nor outward reachability are sufficient on their own. Node Arc in Fig. 2c has highly active paths to a large number of nodes, and is the best candidate for an epicenter.

We formulate a new measure, *ripple centrality*, which serves as a logical AND between closeness centrality and outward reachability.

$$ Ripple\, centrality(u) = C(u)* R_{out}(u) $$

In the calculation, both closeness centrality and outward reachability are normalized. Ripple centrality is calculated for the nodes in the perturbed CSHAN, resulting in the proteins being ranked on the basis of their effectiveness as potential epicenters (Fig. 1(d)). The ranked list is then split into two lists – (a) nodes occurring only in the perturbed CSHAN, and (b) nodes common to both CSHANs (Fig. 1(e)). Common nodes work as global epicenters, playing key roles both before and after the perturbation. Since identical paths have already been discarded (Fig. 1(b)), these proteins are those which have undergone re-wiring, and participate in a different pathway upon perturbation. The nodes occurring only in the perturbed CSHAN are epicenters specific to the perturbation, involved in either the spread of the perturbation or the reaction to it.

### EpiTracer algorithm

The EpiTracer algorithm consists of three modules (1) *highest_activity_paths* extracts the paths with cost inside a user-defined percentile threshold, (2) *condition_specific_han* uses *highest_activity_paths* to identify the highest activity network specific to each condition, and (3) the main module, *get_epicenters*, uses the above two modules to identify the top 10 epicenters in the perturbed condition, as well as the top 10 epicenters common to both conditions. The pseudocode for each module is provided in Algorithms 1, 2 and 3. The symbols *G*_*A*_ and *G*_*B*_ refer to the graph for condition A and the graph for condition B, respectively.







### Biological analysis

The proteins identified as epicenters, as well as the proteins surrounding them were subjected to biological and functional analysis.

#### Immediate influence zone

The nodes that occur within two hops upstream or downstream from an epicenter are designated the *immediate influence zone* of that epicenter. For the top-ranked epicenter, the immediate influence zone was identified manually and was restricted to the perturbed highest activity network. Downregulated genes which occur within two hops of the epicenter were picked from the full network and added to the influence zone.

Since manually examining the full network for dysregulated genes in the vicinity of every epicenter is a time consuming and laborious task, an automated script was developed to facilitate the quick extraction of the influence zone. This can be done on the full network or on the highest activity network. This allows for easy identification of nodes with significant dysregulation, and can be used for further analysis. This script uses a default fold change cut-off of 2.0. Both the number of hops and the fold change can be varied by the user if necessary.

#### Functional enrichment

Gene set enrichment was performed against the KEGG [12] database using WebGestalt [17]. A hypergeometric test with P-value of 0.05 with FDR correction was used for statistical analysis. Network visualization was carried out with Cytoscape, and the Cytoscape plugin ClueGO [18] was used for GO module enrichment.

### Sensitivity analysis

Two separate sensitivity analyses were carried out, one by increasing the expression levels of all genes by a randomly chosen value between 0 and 5 %, and the other by decreasing the expression levels of all genes similarly. This reflects measurement errors that can be introduced in the microarray data due to variability in the sensitivity of the detector. All numbers reported are an average of 100 independent experiments.

## Results

The algorithm was implemented in Python 2.7, and uses the functions provided by Networkx 1.7 for computing all the centrality measures. Dijkstra ^′^s algorithm [19] was used for finding shortest paths. The EpiTracer algorithm was able to analyse a dataset consisting of 10,306 nodes and 74,404 edges on a 16 core Xeon server in less than 30 minutes.

The results of the first case study are provided in detail in the next section, followed by a summary of the second case study.

### Case study 1

Microarray data for the overexpression of PARK2 in human glioma cell line (U251) and control (GFP) were taken from (E–GEOD–61973) [9]. PARK2 (PARKIN) is an E3 ubiquitin ligase whose dysfunction has been associated with Parkinsonism. The authors of this data, in their study [9], show that PARK2 is frequently deleted or downregulated in human glioma, and demonstrate that overexpression of PARK2 can significantly inhibit glioma cell growth. Through the EpiTracer algorithm, we uncover the global reprogramming of gene expression resulting from this perturbation, and highlight the epicenters of this process. We also provide a ranked list of influential players in this perturbation.

#### System description

**Fig. 3:**
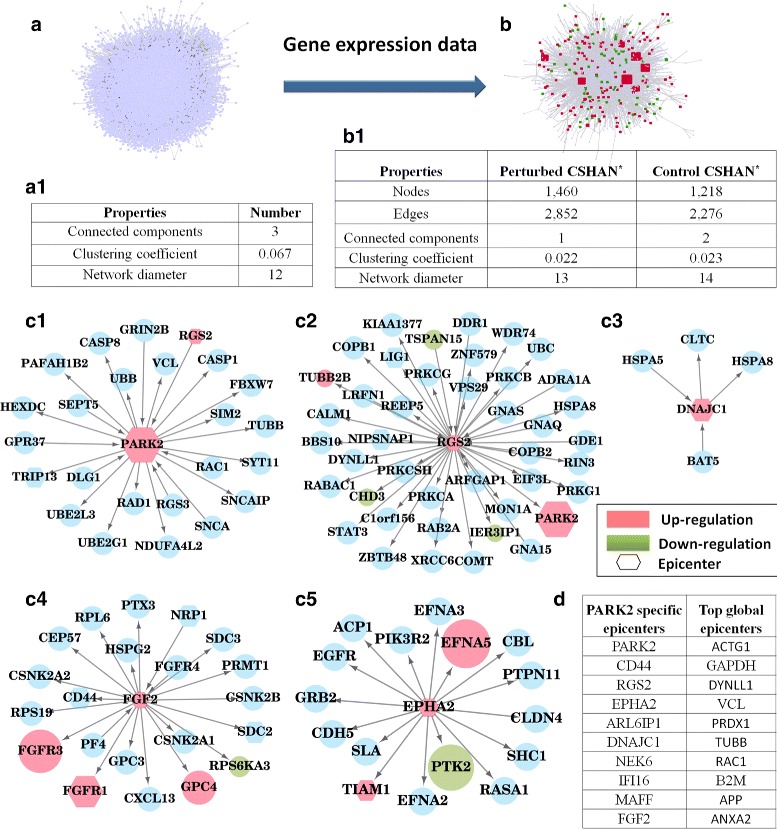
Case study 1 (PARK2 overexpression in human glioma cell line). Data corresponding to overexpression of PARK2 in human glioma cell line (U251). **a** Human PPI network comprising of 10,306 nodes and 74,404 edges. Nodes colored red are upregulated upon perturbation, and nodes colored green are downregulated. (a1) Table of network properties. **b** Perturbation-specific *HAN (highest activity network), with network properties in table b1. **c** The 5 epicenters which were differentially expressed, along with their immediate neighbors **d** List of epicenters specific to the perturbation as well as global epicenters

The gene expression profiles were normalized and filtered, and the list of differentially expressed genes was extracted using a fold change cut-off of 1.5. It was found that 605 genes were downregulated and 1,089 genes were upregulated as a result of the overexpression of PARK2. In general, genes associated with cell cycle, ubiquitin mediated proteolysis, ErbB signaling pathway, MAPK, JAK-STAT signaling, WNT signaling, Hedgehog signaling pathway and pathways related to lipid metabolism were differentially expressed. A summary of network properties is shown in Fig. 3a.

#### Highest activity paths (HAPs)

All-pairs-shortest paths were calculated for the control network as well as the perturbed network. Paths with length ≥ 2 were sorted in the ascending order of path cost. It was found that the number of paths retained at a percentile cut-off of 0.2 was twice that retained when a cut-off of 0.1 was used. Thus the conservative threshold of 0.1 percentile was chosen, resulting in 67,728 paths being retained as *highest activity paths* (HAPs) in the perturbed network and 58,570 HAPs in the control network.

#### Condition-specific highest activity network (CSHAN)

Highest activity paths common to both conditions correspond to the paths which are highly active all the time, and are unaffected by the perturbation. Such paths were removed, giving us 9,621 HAPs specific to the control condition, and 18,779 HAPs specific to the perturbed condition. The edges involved in these paths correspond to the condition-specific highest activity networks (CSHANs). Interestingly, the CSHANs were themselves well-connected networks (Fig. 3b).

Of the 1,756 genes in the perturbed CSHAN, 75 genes were found to be downregulated, and 130 were found to be upregulated. These belonged to the functional categories of cell cycle, MAPK, ErbB, p53 and mTOR signaling pathway, ubiquitin mediated proteolysis, regulation of actin cytoskeleton and oocyte meiosis.

#### Tracing the epicenter

The nodes in the perturbed CSHAN were ranked in descending order of their ripple centrality. This ranked list was then split into two - nodes occurring only in the perturbed CSHAN, and nodes common to both CSHANs (global epicenters). Since common paths have already been removed, nodes common to both CSHANs correspond to the nodes which participate in a different pathway after the perturbation. Nodes occurring only in the perturbed CSHAN are those which have become active and influential after the perturbation.

The top 10 nodes from each list were considered as epicenters, and are listed in Fig. 3d. PARK2 was identified as the highest ranked epicenter among the nodes unique to the perturbed CSHAN, in spite of the fact that the algorithm was given no prior knowledge of the perturbation. Only 5 out of the 10 epicenters specific to the perturbed condition were found to have significant differential expression. This shows that EpiTracer is able to capture information that simple differential expression analysis cannot. The 5 epicenters which were differentially expressed, along with their immediate neighbors, have been depicted in Fig. 3c.

#### Biological interpretation

Top global epicenters were found to correspond to highly conserved and ubiquitously expressed proteins such as TUBB, GAPDH, VCL, ACTG1, DYNLL1 and ANXA2. RAC1 is known to promote cell migration and invasion in glioma cells. APP is associated with axonogenesis, neurite growth and neuronal adhesion [20]. PRDX1 is involved in redox regulation of the cell. B2M is associated with MHC Class I antigen presentation.

Further, the top epicenters specific to the perturbed (PARK2 overexpression) condition were examined. It was found that 5 out of the 10 genes being examined showed significant differential expression, namely PARK2, RGS2, EPHA2, DNAJC1 and FGF2 (Fig. 3c). PARK2 was highlighted as the most important epicenter specific to the PARK2 overexpression condition. PARK2 negatively regulates cell cycle by degrading Cyclin E and D through its activity as an E3 ubiquitin ligase. RGS2 is involved in G0 to G1 transition [20]. Inhibition of EPHA2 leads to stalling of cells in G0/G1 phase [21]. In the PARK2 overexpression condition, EPHA2 was found to be upregulated. FGF2 blocks cell proliferation and causes a G2/M arrest [22]. When considered together, our analysis revealed that most of the top ranked genes were associated with cell cycle regulation.

##### Immediate influence zone of the top-ranked epicenter

**Fig. 4:**
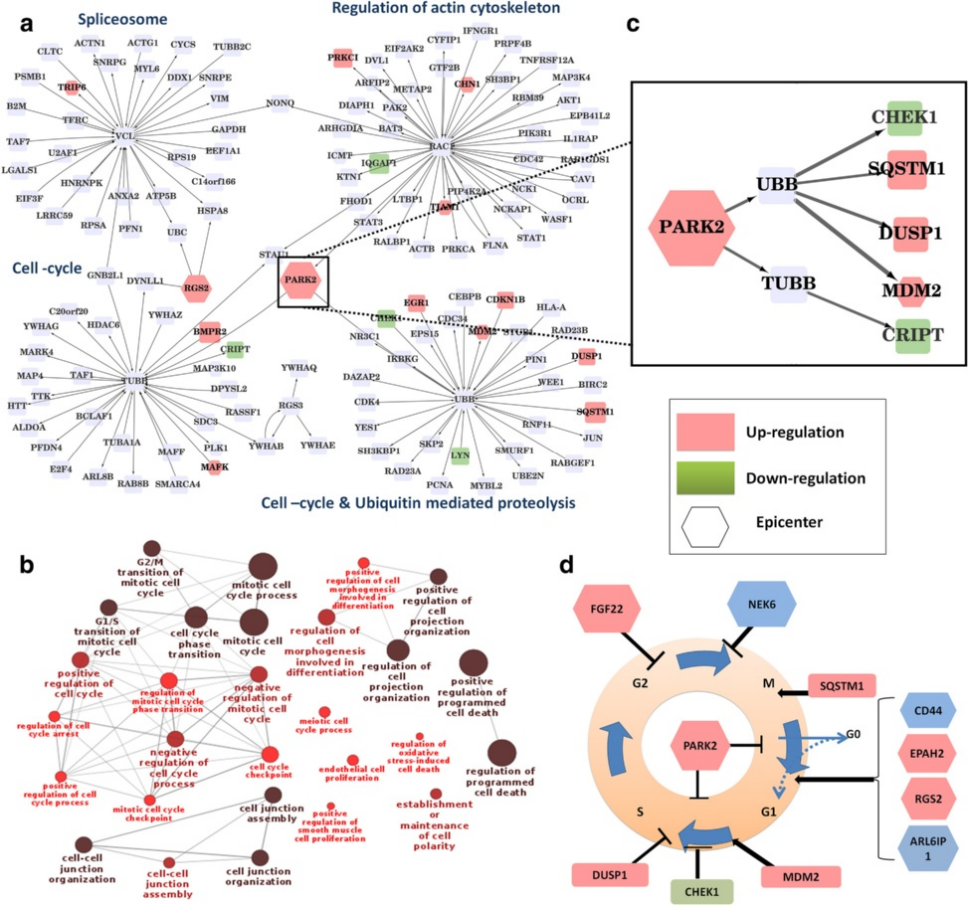
PARK2 influence zone. Detailed biological interpretation of PARK2 influence zone. **a** The PARK2 influence zone consists of 118 nodes and 119 edges. Red colored nodes correspond to upregulated genes, and green corresponds to downregulated genes. The epicenter is depicted using a hexagon. **b** GO enrichement of genes in PARK2 influence zone shows that most genes are involved in cell-cycle regulation. **c** Nodes downstream of PARK2 **d** Mechanistic insights into cell-cycle dysregulation upon PARK2 overexpression

In order to understand the cellular response to the top-ranked epicenter specific to the perturbed condition (PARK2 in this case), the influence zone around it was analysed. The subgraph induced by considering nodes upto two hops up/downstream of PARK2 in the perturbed CSHAN were considered to be in the PARK2 influence zone. Any downregulated nodes within 2 hops of PARK2 in the complete network were also added (Fig. 4a). GO enrichment was carried out specifically for cell cycle regulation as PARK2 is known to be a cell cycle regulator. Interestingly, it was found that the PARK2 influence zone was highly enriched for cell cycle regulation (Fig. 4b), including G2/M transition and G1/S transition of mitotic cell cycle, mitotic cell cycle, positive and negative regulation of cell cycle.

The influence exerted by PARK2 was studied by focusing on the nodes downstream of PARK2 (Fig. 4c). It was found that many downstream genes such as MDM2, CHEK1, SQSTM1 and DUSP1 were involved in cell cycle regulation.

Since overexpression of PARK2 inhibits the progression of cell cycle, the expected response from the cell would be to modify other regulatory mechanisms of cell cycle progression to counteract this arrest. Examination of the nodes downstream of the top-ranked epicenter (PARK2) showed that this was indeed the case (Fig. 4d). Major remodeling can be inferred from the G0/G1 and G1/S transition. SQSTM1 (P63) is involved in exiting of the cell from the M phase in the cell cycle. CD44, EPHA2, RGS2 and ARL6IP1 are positive regulators for G0/G1 transition. MDM2 is an activator of G1/S transition as it inhibits P53 and Rb proteins. However, CHEK1 and DUSP1 are repressors of G1/S phase transition. CHEK1 acts as a Cyclin E repressor by inhibiting Cdc at the DNA-repair check-point. DUSP1 is a repressor of the MAPK pathway [23]. FGF2 and NEK6 are repressors of G2/M phase transition [24].

Since creation of such influence zones for every highly ranked gene is a tedious task, an automated script was developed to output the influence zone as well as to summarize the details of differentially expressed genes in an easy-to-read table. The table thus generated for the second highest ranked epicenter, CD44, is shown in Table 1.

**Table 1:** Automated summary for CD44 (case study 1). CD44 was the 2 ^*nd*^ ranked epicenter specific to the perturbed condition. The table shows the nodes in the immediate influence zone (up to 2 hops up/downstream) of CD44 which showed significant differential expression (2-fold). The first row corresponds to the input node, CD44. In the subsequent rows, the first column shows the differentially expressed gene (DEG). If the DEG is more than 1 hop away from CD44, the intermediate nodes on the unweighted shortest path are described in columns 6 onwards

Node	Direction	Num_hops	Fold_change	Which_network	Intermediate_node_1	Fold_change	Significant_fc?	Which_network
CD44	input_node	0	1.0776225906	unique to perturbed CSHAN				
L1CAM	down_CD44	2	4.3995043553	unique to perturbed CSHAN	EZR	1.4077313131	False	common to both CSHANs
CBLB	down_CD44	2	2.7450193138	unique to perturbed CSHAN	EGFR	0.9625043727	False	common to both CSHANs
TNNT1	down_CD44	2	0.3519677136	unique to control CSHAN	FYN	1.047836048	False	common to both CSHANs
RPS6KA2	down_CD44	2	3.7937197295	unique to perturbed CSHAN	EGFR	0.9625043727	False	common to both CSHANs
NEDD9	down_CD44	2	0.4699818669	not in any CSHAN	FYN	1.047836048	False	common to both CSHANs
TFPI	down_CD44	2	2.7743880699	not in any CSHAN	MMP7	0.1323159054	True	not in any CSHAN
MBNL3	down_CD44	2	4.0574758277	not in any CSHAN	LCK	0.967172212	False	not in any CSHAN
IVNS1ABP	down_CD44	2	2.0134951539	common to both CSHANs	ARHGEF1	1.0053092034	False	not in any CSHAN
ITGB3	down_CD44	2	0.2450269938	not in any CSHAN	COL1A2	1.6105271705	False	common to both CSHANs
PLA2G4A	down_CD44	2	7.496384226	not in any CSHAN	COL1A2	1.6105271705	False	common to both CSHANs
FN1	down_CD44	2	2.2964207553	unique to perturbed CSHAN	COL1A2	1.6105271705	False	common to both CSHANs
MEF2C	down_CD44	2	2.2667831761	unique to perturbed CSHAN	CD74	0.9449408306	False	not in any CSHAN
PTK2	down_CD44	2	0.2821339725	not in any CSHAN	EGFR	0.9625043727	False	common to both CSHANs
CHN1	down_CD44	2	3.1958019401	unique to perturbed CSHAN	TGFBR1	0.7284950123	False	common to both CSHANs
EGR1	down_CD44	2	2.1681746909	common to both CSHANs	ARHGEF1	1.0053092034	False	not in any CSHAN
SRGN	down_CD44	1	0.4947740703	not in any CSHAN				
OCLN	down_CD44	2	2.9381790192	not in any CSHAN	TGFBR1	0.7284950123	False	common to both CSHANs
ADAM12	down_CD44	2	2.3217184015	unique to perturbed CSHAN	IGFBP3	1.9432883668	False	common to both CSHANs
TIMP1	down_CD44	2	0.4692561311	unique to control CSHAN	MMP1	0.9852575467	False	not in any CSHAN
MMP7	down_CD44	1	0.1323159054	not in any CSHAN				
L1CAM	up_CD44	2	4.3995043553	unique to perturbed CSHAN	ANK1	0.7930398677	False	not in any CSHAN
ITGB3	up_CD44	2	0.2450269938	not in any CSHAN	COL1A2	1.6105271705	False	common to both CSHANs
MMP7	up_CD44	1	0.1323159054	not in any CSHAN				

#### Sensitivity analysis

The gene expression levels of all the genes were either increased or decreased as indicated in the Methods section. The results of the 200 independent runs were then analysed to check how the top ranked epicenters fared. It was found that 9 nodes were always present in the top 10 ranked epicenters specific to the perturbed condition irrespective of the direction or extent of perturbation. When the top 20 ranks were considered, 16 nodes were common to all 200 experiments. Also, PARK2 was ranked the 9.6^*th*^ most important epicenter specific to the perturbed condition on average out of 10,306 possible candidates. This shows that even when every single node in the network was perturbed, the nodes ranked as epicenters remained largely unaffected.

### Case study 2

Microarray data for the knockdown of SP1 gene in HeLa cells were taken from GSE37935 [10]. The knockdown was carried out by treating HeLa cells with an siRNA directed against the SP1 mRNA. SP1 is a global transcription factor, and regulates various important biological processes such as proliferation, cell differentiation and oncogenesis. Since the knockdown of a transcription factor can lead to downregulation of its target genes which are positively regulated, these genes will have higher activity in the control condition. Hence in this scenario, we analyse epicenters specific to the perturbed as well as the control condition.

#### Biological interpretation

In the perturbed (SP1 knockdown) condition, the top 10 ranked epicenters consist of 14 genes. 5 genes are assigned the same rank due to similar activity and connectivity. Out of the 14 epicenters, 5 genes, namely GPRC5A, EBF1, PTPN4, FAS and ADCK2 were differentially expressed. GPRC5A, EBF1 and PTPN4 genes play important roles in development, cellular growth, and differentiation [20]. FAS is involved in physiological regulation of programmed cell death. The function of ADCK2 is not yet clear. In the control condition, top 10 epicenters include 50 genes, with 30 genes being ranked 7^*th*^ and 9 genes being ranked 2 ^*nd*^ due to similar activity. In this case, SP1 appeared as the 10^*th*^ ranked epicenter.

##### Immediate influence zone

**Fig. 5:**
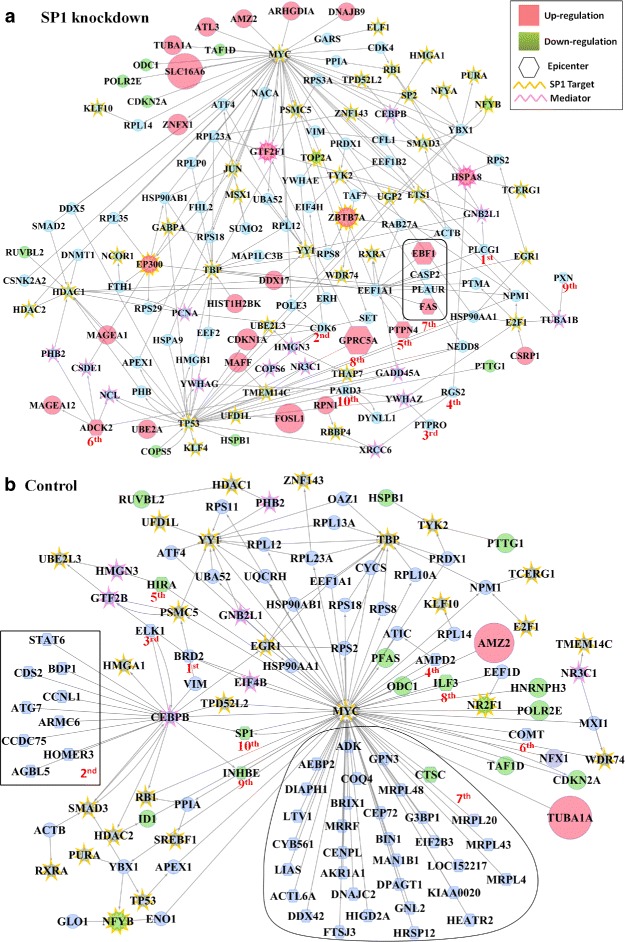
Case study 2 (SP1 knockdown in HeLa cell line). Influence zone of the top 10 epicenters was constructed from the condition-specific highest activity network and enriched with the targets of SP1 and their immediate neighbors. This network was pruned to retain only epicenters, SP1 targets, differentially expressed genes, and the genes connecting them. Nodes with a hexagonal shape represent epicenters, a golden border around the node indicates SP1 target, and a pink border around the node indicates mediator gene. The rank of each epicenter is written next to it in red. (**a**) SP1 knockdown condition. 14 genes occur in the list of top 10 epicenters (5 genes correspond to rank 5). (**b**) Control condition. 50 genes correspond to top 10 epicenters. 30 genes correspond to rank 7, and regulate MYC, a target of SP1. Similarly, 9 genes correspond to rank 2, and regulate CEBPB

The immediate influence zone of the top 10 epicenters was constructed as a combined network. The targets of SP1 and their first interactors were added to this network, and the entire network was pruned to retain only epicenters, targets of SP1, differentially expressed genes, and genes which were essential for the connectivity of the graph. This pruned graph contains 142 nodes and 228 edges, and is shown in Fig. 5a. Analysis of the graph showed that epicenters were generally indirect regulators of the targets of SP1. This could indicate that alternative methods of regulating SP1 targets gained importance due to the knockdown of SP1. Many targets of SP1 were found in the highest activity paths which trace back to the epicenters. For example, MYC and TP53 were highlighted as important genes regulated by SP1, and 10 regulators of MYC were ranked as epicenters, with 5 of them being assigned the same rank due to similar activity. The paths tracing back to the epicenters clearly illustrate the cascade of influence of the epicenters to the targets of SP1, involving mediator genes. The most prominent mediator genes in the SP1 knockdown condition are EEF1A1 and HSPA8. EEF1A1 is regulated by 7 epicenters, of which FAS, EBF1 and PTPN4 are differentially expressed. EEF1A1 in turn regulates 5 targets of SP1, of which EP300 is differentially expressed. EP300 is a transcriptional co-activator protein, and is important in the processes of cell proliferation and differentiation.

Since the data being analysed is of knockdown of a transcription factor (SP1), we investigate the targets regulated by SP1 by focusing on the highest activity network specific to the control condition. The influence zone for the epicenters with top 10 ranks was constructed and pruned as in the perturbed condition. This graph contains 125 nodes and 168 edges, and is shown in Fig. 5b. MYC and CEBPB emerged as important genes in this condition. MYC is a direct target of SP1, and also regulates other targets of SP1. CEBPB is an important mediator gene, which regulates 4 targets of SP1, and is regulated by 9 epicenters, all of which were ranked 2 ^*nd*^.

In conjunction, the analysis of the two conditions revealed that the effect of SP1 knockdown spreads through 3 important hubs - MYC, CEBPB and TP53. Regulators of these important hubs were ranked as epicenters by our algorithm. MYC has 11 target genes which are differentially expressed. ZNFX1, TAF1D, NFX1, TFIIF and NFX1 are involved in tanscriptional and post transcriptional regulation [20]. CDKN2A activity leads to cell cycle arrest. ODC1 is an enzyme of polyamine metabolism and PFAS participates in purine metabolism [12]. Both metabolic pathways are necessary for DNA replication and transcription. NFX1 is mainly involved in inflammatory response. TP53 also regulates 11 genes, of which three genes, namely PTTG1, COPS5 and CDKN2A, are differentally expressed. COPS5 is one of the members of the COP9 signalosome which regulates mutiple signaling pathways [20]. PTTG1 is involved in cell cycle regulation. CEBPB regulates 10 gene in the control condition, of which two were differentally expressed - SP1 and INHBE.

## Discussion

EpiTracer identifies nodes at which highly active paths originate and which are able to reach a large fraction of the active network. When annotated with the condition in which they are active, these nodes correspond to the most influential players in that specific condition and are termed epicenters. It is important to note that the epicenter does not necessarily correspond to the source of the perturbation.

EpiTracer can be expected to have wide applicability, demonstrated here by two entirely different datasets studied in this work. Since the algorithm focuses on active nodes and edges, the network on which the analysis is carried out must be chosen based on the context. As demonstrated in case study 1, analysing the perturbed highest activity network is preferable when the perturbation is expected to be an upregulation event. If the perturbation is expected to be a downregulation event, analysing the control highest activity network will yield the set of nodes which were influential before the knockdown (case study 2). An analysis of the perturbed highest activity network is also useful since it can yield a list of epicenters that are activated in the perturbed condition upon removal of the knocked-out regulator. If the nature of the perturbation is unknown, both highest activity networks should be analysed. A limitation of the algorithm is that the source of the perturbation may not appear in the highest activity networks if its expression level remains low both before and after the perturbation. In such cases EpiTracer will be able to highlight the highly active nodes close to the source of the perturbation, but not the source itself.

It was observed during the course of this work that the largest strongly connected component (LSCC) plays an important role in spreading a perturbation through the network. The largest strongly connected component is the largest subgraph in which there exists a path from every node to every other node. It was found that the epicenter was a part of the LSCC in the highest activity network under study. If the LSCC comprises a big enough percentage of the graph, we believe it might be possible to speed up the algorithm by restricting the search only to the nodes in the LSCC.

## Conclusion

We propose a new algorithm, EpiTracer, to trace the epicenter of perturbations in a condition-specific biological network. The algorithm is capable of extracting the highest activity network specific to each condition under study and ranking the nodes in these highest activity networks with a ripple centrality score, which reflects how well any influence from that node can ripple out into the rest of the network.

The algorithm has been demonstrated on two case studies, one where a gene was overexpressed, and another where a gene was knocked down. In the case of overexpression, EpiTracer was able to identify the overexpressed gene as the most important epicenter. Biological analysis of the top-ranked epicenters showed that all of them had functions relevant to cell cycle progression, and highlighted a scenario where the most important epicenters were involved in either spreading the influence of PARK2 or working to counteract its effect. Also, 5 of the top 10 epicenters showed no significant change in expression level, and yet were found to be biologically meaningful epicenters. This shows that our algorithm is able to highlight more than simple differential expression. The immediate influence zone of PARK2 generated by the EpiTracer pipeline, and the dysregulated genes in this were also found to be enriched in genes involved in cell cycle regulation. In the knockdown case study, alternative regulators of the knocked-down gene’s targets were highlighted as epicenters. Also, the gene that was knocked down was picked up as an epicenter in the control condition. This demonstrates the general applicability of the algorithm. Sensitivity analysis has been carried out to show that the epicenters identified by EpiTracer are largely unaffected by small changes in the network.

The EpiTracer algorithm identifies the epicenters which either spread a perturbation or respond to it. The paths along which the influence ripples out of the epicenters is highlighted by the condition-specific highest activity network. This gives a system-wide, unbiased view of a disease phenotype, and how the organism responds to it.
